# Uncertainty analysis on flood routing of embankment dam breach due to overtopping failure

**DOI:** 10.1038/s41598-023-47542-6

**Published:** 2023-11-17

**Authors:** Miaofan Yang, Qian Cai, Zhuo Li, Jiangui Yang

**Affiliations:** 1 Hohai University, Nanjing, 210024 China; 2 Nanjing Hydraulic Research Institute, Nanjing, 210029 China; 3 Management Office of Nanjing Sancha River Estuary Sluice, Nanjing, 210036 China

**Keywords:** Natural hazards, Civil engineering

## Abstract

The breach risk of reservoir dam exists objectively while exerting benefits. There is great uncertainty in the overtopping breach process of embankment dam and the dam break flood routing which affects the accuracy of dam risk analysis and may cause unnecessary waste in emergency scheduling. However, the uncertainty analysis of the breach process and its consequence is currently inadequate. Therefore, a stratified sampling Monte Carlo method is proposed to simulate uncertain overtopping breach flood of embankment dams. The main sources of uncertainty are analyzed and determined as uncertain dam breach and flood routing processes. The uncertain breach process of dam is studied by presenting a sensitive study between 3 mechanism breach models and 5 parametric breach models. The random dam breach process is restored using HEC-RAS semi-empirical breach model by estimating breach characteristics through multiple common breach models. The random flood routing is carried out through 1D–2D coupled unsteady flow analysis in which the random breach process is adopted as an upper inflow boundary condition. According to the case study results, though parameters have been controlled in a limited range, the flood routing results in the early stage of dam overtopping failure present greater uncertainty. As the flood progresses further downstream, the uncertainty will gradually decrease. This study could serve as a reference for dam breach risk map making.

## Introduction

Reservoir plays an important role in flood control and drought resistance. However, it also poses a potential risk to the downstream. By the end of 2020, there were 3369 dam-breaks in China, of which 1737 were caused by overtopping, accounting for 51.7% of the total. Most of them were embankment dams^1^. The risk of overtopping failure of embankment dams is widely concerned by the industry.

A series of achievements have been made on the mechanism, simulation, probability, consequence and flood routing of overtopping failure of embankment dam^2^. Relevant specifications of flood risk mapping for dam breach have been documented and flood risk maps have been widely produced^3^. However, there are great uncertainties in reservoir inflow process, dam breach process and flood routing process, which affect the accuracy of risk analysis^4^. As a result, unnecessary waste in emergency dispatching may be caused by inaccurate dam breach flood analysis.

At present, researches on the failure mechanism of embankment dams are relatively comprehensive and in-depth^5,6^. Many mature numerical simulation models for dam failure have been developed. For example, BREACH^7^, DAMBRK^8^, MIKE DB^9^, Chen Shengshui Model^10^, HEC-RAS Model^11^, BRDAM^12^, IWHR DB^13^, BEED^14^, etc., have been widely adopted at home and abroad. The models above are all deterministic analysis models. The uncertainty of the model results is mostly analyzed from the aspects of sensitivity^15^. However, sensitivity analysis could not consider the probability distribution of the stochastic variables. And the variables have not been considered in actual reasonable parameter ranges. Therefore, the influence of individual parameter uncertainty on model results may be misestimated. The stochastic process analysis and uncertainty simulation of dam overtopping failure are relatively inadequate.

On the other hand, as the dam breach risk is generally defined as the product of dam breach probability and dam breach consequence, the results of uncertain factors are mostly concerned in the probability of dam breach. The uncertainty of the dam breach consequences is seldom considered, and the related uncertainty researches mostly start from the aspects of various of losses caused by dam breach^16^. For example, Qi et al. studied the randomness of property distribution and loss rate curve in flood risk assessment^17^. De Bruijn et al. used Monte Carlo method to evaluate the annual expected life loss and draw the probability curve of life loss by sampling the maximum flow, maximum water depth, location of the outburst and population refuge ratio during the typical return period^18^. Jiao et al. proposed a new fuzzy model to sequence the life loss risk consequences caused by dam failure where the relative difference formula was improved through logarithmic transformation and boundary constraint^19^. In fact, due to a variety of uncertainties, the simulation results of flood routing, which determine the consequences of dam breach, have also significant uncertainty. Mazzoleni et al. drew an uncertain flood risk map considering the uncertainty of location, geometry, and the formation process of the levee piping breach^20^. Bates et al. studied the uncertainty of flood routing model based on grid simulation, in which the un certainty effect of Manning roughness parameter on the water level of typical cross section in flooded area is mainly analyzed^21^. However, the above study is not coupled with the uncertainty of dam breach discharge process. In general, the research on the uncertainty analysis of flood routing caused by overtopping failure of embankment dam is relatively inadequate. The reliability of dam breach risk analysis needs to be further studied.

In this paper, the uncertainty in the discharge process of overtopping flood is analyzed. The stochastic process of overtopping dam breach is simulated by restoring to various breach models. And the reliability of flood risk mapping is verified through an engineering example which could solidate the dam risk management foundation and serve as a technical support and reference for similar projects.

## Uncertain flood routing analysis method for overtopping failure of embankment dam

### General method of flood routing simulation for overtopping failure of embankment dam

The flood routing for overtopping failure of embankment dam generally contains two steps of simulations: the overtopping failure simulation of embankment dam and the flood routing simulation.

#### Overtopping breach models of embankment dam

The dam breach model is a technique to estimate the breach parameters (shape, height, width, final forming time, etc.) and then predict the discharge process which could be divided into mechanism model, parametric model and semi-empirical model.

Mechanism models are established due to the physical mechanism of the dam failure process based on the historical dam failure cases and laboratory tests. Those models commonly involve flow routing model, erosion model and breach expansion model. For example, BREACH, DAMBRK, MIKE DB, IWHR DB, etc. Their main erosion models served as governing equations are as follows:

1 $$ \left\{ \begin{gathered} q_{b} = 3.64\left( {\frac{{d_{90} }}{{d_{30} }}} \right)^{0.2} P\frac{{D^{2/3} }}{n}S^{1.1} (DS - \Omega ) \hfill \\ \Omega = 0.005\tau_{c} d_{50} {\text{ (cohesionless soil)}} \hfill \\ \Omega = \frac{{b^{\prime}}}{62.4}(PI)^{{c^{\prime}}} {\text{ (cohesive soil}}) \hfill \\ \Delta H_{c} = 3600\Delta tq_{b} /[PL(1 - n_{0} )] \hfill \\ \end{gathered} \right.\quad ({\text{BREACH}}) $$

2 $$ \left\{ {\begin{array}{*{20}l} {b_{b} = b(t_{b} /t)} \hfill & {0 < t_{b} < t} \hfill \\ {h_{b} = h_{d} - (h_{d} - h)\frac{{t_{b} }}{t}} \hfill & {0 < t_{b} < t} \hfill \\ \end{array} } \right.\quad ({\text{DAMBRK}}) $$

3 $$ \left\{ \begin{gathered} q_{s} = 0.05V^{2} \left( {\frac{{d_{50} }}{g(s - 1)}} \right)^{0.5} \left( {\frac{\tau }{{\rho g(s - 1)d_{50} }}} \right)^{1.5} \hfill \\ \frac{dh}{{dt}} = \frac{{q_{s} }}{L(1 - \varepsilon )} \hfill \\ \frac{db}{{dh}} = 2x \, \quad \, x \in [0.5,1] \hfill \\ \end{gathered} \right.\quad ({\text{MIKE DB}}) $$

4 $$ \left\{ \begin{gathered} \frac{\Delta z}{{\Delta t}} = \Phi (\tau ) = \frac{v}{a + bv} \hfill \\ v = k(\tau - \tau_{c} ) \hfill \\ \end{gathered} \right.\quad ({\text{IWHR DB}}) $$

where *q*_*b*_ is discharge per unit width; *d*_30_, *d*_50_, *d*_90_ are respectively particle sizes accounting for 30%, 50% and 90% of soil mass; *P* is wetted perimeter; *D* is breach water level;* n* is roughness coefficient;* S* is inverse of downstream slope; *τ*_*c*_ is critical shear stress; *PI* is plastic index; ∆*H*_*c*_ is erosion depth per unit time; *L* is erosion length of breach channel; *n*_0_ is void ratio; *b*′,* c*′ are empirical coefficients; *b*_*b*_ is breach bottom width at time *t*_*b*_; *b* is final breach bottom width; *t* is total breach time;* h*_*b*_ is breach bottom elevation at time *t*_*b*_;* h*_*d*_ is breach depth at time *t*_*b*_ determined by a regression equation;* h* is final breach bottom elevation; *q*_*s*_ is sediment transport rate; *V* is flow velocity; *s* is soil relative density; *τ* is shear stress; *ρ* is water density;* h* is breach depth; *b* is breach width; *L* is erosion length of breach channel; *z* is erosion depth; *v* is shear stress deducting the critical shear stress; *k* is unit transformation factor; *a*,* b* are empirical coefficients.

Parametric model refers to the establishment of a database by collecting the breach parameters of historical dam failure cases. And the regression analysis is adopted to deduce the parameter equations of the final breach parameters and related characteristics of reservoir capacity, dam height, reservoir water level, etc. The more famous ones are MacDonald equation^22^, Froehlich equation^23,24^, Von Thun and Gillete equation^25^, Xu and Zhang equation^26^, etc. The regression equations for the above parametric models are as follows:

5 $$ \left\{ \begin{gathered} V_{eroded} = \left\{ \begin{gathered} 0.0261(V_{out} \cdot h_{w} )^{0.769} {\text{ (for earthfill dams)}} \hfill \\ 0.00348(V_{out} \cdot h_{w} )^{0.852} {\text{ (with clay core or rockfill dams)}} \hfill \\ \end{gathered} \right. \hfill \\ t_{f} = 0.0179(V_{eroded} )^{0.364} \hfill \\ W_{b} = \frac{{V_{eroded} - h_{b}^{2} (CZ_{b} + h_{b} Z_{b} Z_{3} /3)}}{{h_{b} (C + h_{b} Z_{3} /2)}} \hfill \\ \end{gathered} \right.\quad ({\text{MacDonald}}) $$

6 $$ \left\{ \begin{gathered} B_{ave} = 0.1803K_{0} V_{w}^{0.32} h_{b}^{0.19} \hfill \\ t_{f} = 0.00254V_{w}^{0.53} h_{b}^{ - 0.90} \hfill \\ \end{gathered} \right.\quad ({\text{Froehlich}},{1995}) $$

7 $$ \left\{ \begin{gathered} B_{ave} = 0.27K_{0} V_{w}^{0.32} h_{b}^{0.04} \hfill \\ t_{f} = 63.2\sqrt {\frac{{V_{w} }}{{gh_{b}^{2} }}} \hfill \\ \end{gathered} \right.\quad ({\text{Froehlich}},{2}00{8}) $$

8 $$ \left\{ \begin{gathered} B_{ave} = 2.5h_{w} + C_{b} \hfill \\ t_{f} = \left\{ \begin{gathered} \frac{{B_{ave} }}{{4_{{h_{w} }} }}{\text{ (erosion resistant)}} \hfill \\ \frac{{B_{ave} }}{{4_{{h_{w} }} + 61.0}}{\text{ (easily erodible)}} \hfill \\ \end{gathered} \right. \hfill \\ \end{gathered} \right.\quad ({\text{Von Thun and Gillete}}) $$

9 $$ \left\{ \begin{gathered} \frac{{B_{ave} }}{{h_{b} }} = 0.787\left( {\frac{{h_{d} }}{{h_{r} }}} \right)^{0.133} \left( {\frac{{V_{w}^{0.32} }}{{h_{w} }}} \right)^{0.652} e^{{B_{3} }} \hfill \\ \frac{{B_{t} }}{{h_{b} }} = 1.062\left( {\frac{{h_{d} }}{{h_{r} }}} \right)^{0.092} \left( {\frac{{V_{w}^{1/3} }}{{h_{w} }}} \right)^{0.508} e^{{B_{2} }} \hfill \\ \frac{{t_{f} }}{{t_{r} }} = 0.304\left( {\frac{{h_{d} }}{{h_{r} }}} \right)^{0.707} \left( {\frac{{V_{w}^{1/3} }}{{h_{w} }}} \right)^{1.228} e^{{B_{5} }} \hfill \\ \end{gathered} \right.\quad ({\text{Xu and Zhang}}) $$

where *V*_*eroded*_ is volume of material eroded from the embankment dam; *V*_*out*_ is volume of water that passes through the breach; *h*_*w*_ is water depth above the dam breach bottom;* t*_*f*_ is breach formation time; *W*_*b*_ is bottom width of the breach; *C* is crest width of the dam; *Z*_3_ = *Z*_1_ + *Z*_2_; *Z*_1_ is average slope of the upstream face of dam; *Z*_2_ is average slope of the downstream face of dam; *B*_*ave*_ is average breach width; *K*_*0*_ is constant (1.4 for overtopping failures, 1.0 for piping); *V*_*w*_ is reservoir volume above the breach bottom at time of failure; *h*_*b*_ is final breach height; *g* is gravitational acceleration; *C*_*b*_ is a reservoir size coefficient; *h*_*d*_ is height of the dam; *h*_*r*_ is reference dam height taken as 15 m; *B*_*t*_ is breach top width; *t*_*r*_ is 1 h (unit duration time);*B*_2_, *B*_3,_ *B*_5_ are coefficients representing function of dam properties.

**Figure 1:**
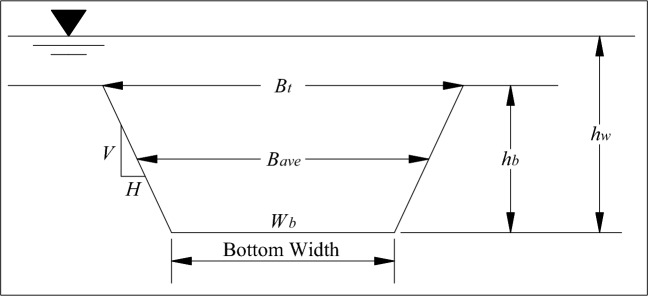
Description of breach parameters required from HEC-RAS.

The parametric model often lacks sufficient theoretical basis, and the mechanism model often has applicability problems because the physical mechanism of water and sediment balance of dam breach has not been completely studied. Therefore, there are also semi-empirical models combining mechanism model and parametric model. HEC-RAS presents a parametric breach model in which a headcut erosion process with a trapezoidal breach is determined by estimating the breaching characteristics. The physical description of the breach will consist of the height of the breach, breach width, and side slopes in H:V. These values represent the maximum breach size. A diagram describing the breach defined by HEC-RAS is shown in Fig. 1.

When performing a dam breach analysis, the characteristics of the breach (shown in Fig. 1) must first be estimated using parametric breach models. The breach width is described as the average breach width (*B*_*ave*_) in many equations, while HEC-RAS requires the breach bottom width(*W*_*a*_) for input. The breach height (*h*_b_) is the verticalextent from the top of the dam to the average invert elevation of the breach. Many publications and equations also use the height of the water (*h*_*w*_), which is the vertical extentfrom the maximum water surface to the invert elevation of the breach. The side slopes areexpressed in units of distance horizontal to every one unit in the vertical (*H*: 1* V*). The breach dimensions, as well as the breach formation time must be estimated outside of the HEC-RAS software, and entered into the program. Once the breaching characteristics are estimated, then HEC-RAS can be used to compute the outflow hydrograph from the breach by defining several breach initial and boundary conditions like max possible bottom width, min possible bottom elevation, starting notch width, et.al. As the breaching characteristics could be estimated through all types of breach models, and the calculation accuracy of HEC-RAS has been verified through many example applications^11^, the HEC-RAS breach model could also be adopted to restore breach processes predicted by various breach models.

#### Mathematical model of two-dimensional unsteady flow for dam breach flood analysis

##### Governing equations

The dam breach flood wave movement in the downstream river channel of a reservoir is a non-constant flow. The relationship between hydraulic elements and space and time could be expressed by the two-dimensional equations of non-constant flow, namely the Saint–Venant equations:

10 $$ \left\{ \begin{gathered} \frac{\partial z}{{\partial t}} + \frac{\partial }{\partial x}(hU) + \frac{\partial }{\partial y}(hV) = 0 \hfill \\ \frac{\partial U}{{\partial t}} + U\frac{\partial U}{{\partial x}} + V\frac{\partial U}{{\partial y}} + g\frac{\partial z}{{\partial x}} + g\frac{{u\sqrt {u^{2} + v^{2} } }}{{c^{2} h}} = v_{t} \left( {\frac{{\partial^{2} u}}{{\partial x^{2} }} + \frac{{\partial^{2} u}}{{\partial y^{2} }}} \right) \hfill \\ \frac{\partial V}{{\partial t}} + U\frac{\partial V}{{\partial x}} + V\frac{\partial V}{{\partial y}} + g\frac{\partial z}{{\partial y}} + g\frac{{v\sqrt {u^{2} + v^{2} } }}{{c^{2} h}} = v_{t} \left( {\frac{{\partial^{2} v}}{{\partial x^{2} }} + \frac{{\partial^{2} v}}{{\partial y^{2} }}} \right) \hfill \\ \end{gathered} \right. $$

where *h* is the water depth; *U* is the x direction velocity; *V* is the y direction velocity; *z* is the water level;* g* is gravitational acceleration;* u* is the vertical average velocity components in x direction;* v* is the vertical average velocity components in y direction; *v*_*t*_ is the turbulent viscosity coefficient; *c* is the Chezy coefficient.

##### Initial conditions

The initial condition for dam-break flood analysis refers to the initial water level of the breach and the calculation region at time t = 0:

11 $$ \left. h \right|_{t = 0} = h_{0} (x,y,0) $$

##### Boundary conditions

The boundary conditions include the land boundary and hydraulic boundary. The land boundary is the boundary where there is no water exchange with the outside region:

12 $$ \frac{\partial Q}{{\partial x}} = 0,\frac{\partial Q}{{\partial y}} = 0 $$

The hydraulic boundary is the boundary where there is water exchange with the outside region:

13 $$ \frac{\partial Q}{{\partial x}} = q_{x} ,\frac{\partial Q}{{\partial y}} = q_{y} $$

The inflow hydraulic boundary is the water level or flow process line which could be determined and calculated by the overtopping failure model of embankment dam. The outflow boundary could be the water level and flow relationship curve.

### Stochastic simulation of overtopping dam breach process and flood routing

According to the general method of flood routing simulation for overtopping failure of embankment dam, its uncertainty factors could be classified into the following three categories: the nondeterministic natural inflow process of the reservoir, the nondeterministic dam breach process and the nondeterministic flood routing process.

The upper boundary condition is partly determined by the inflow process of a reservoir. The uncertainty of inflow process originates from the uncertainty of natural inflow. Although there is obvious uncertainty in the inflow process, the worst flood has already been designed through hydrological analysis considering frequency analysis and adverse combination conditions. The uncertain inflow process of a reservoir will not be considered in the uncertainty analysis. Therefore, the uncertainty of breach flood analysis mainly comes from the dam breach simulation and flood routing.

Since there have been a large number of related studies on the parameter sensitivity of common breach models^27^, and the uncertainty of model parameters is actually far less than the influence brought by the uncertainty of different breach models themselves^28,29^, the analysis on breach model uncertainty is focused.

Although different breach mechanisms, different erosion processes and different calculation parameters are adopted in various breach models, the final simulation results of the breach flow process is highly correlated with the following breaching characteristics: the final breach bottom elevation Z, the final breach bottom width b, the breach slope coefficient m, and the breach formation time T. If the general weir flow formula is adopted to calculate the flow, the main uncertainty parameter should be the weir flow coefficient C.

Because the HEC-RAS semi-empirical model could simulate all overtopping breach process by defining the above parameters, it is adopted to restore the calculation results of other breach models, so as to simulate different breach processes and reflect the influence of model uncertainty. The stochastic breach discharge will also be adopted in the downstream flood routing simulation as an upper boundary using HEC-RAS.

### Uncertain flood routing simulation method based on stratified sampling Monte Carlo method

The objective of nondeterministic breach flood routing simulation is to transfer all kinds of flood routing uncertainty parameters to the probability results of flood routing simulation through breach flood simulation model. Monte Carlo method could be a possible way. In order to reduce the probability calculation error, it is necessary to make more samples fall into the interval that has an important impact on the simulation results. Ordinary sampling methods could only achieve this by increasing the sampling times, which will significantly increase the computational burden. When the single simulation of flood routing takes up to several hours, accomplishing the above task will become impossible. Thus, the stratified sampling method is proposed to ensure that more samples are taken in the interval with important influence so as to reduce the number of samples required for simulation accuracy.

When the number of samples in the interval meets the following formula, the error of calculation results is minimized:

14 $$ N_{j} = N\frac{{d_{j} \sigma_{j} }}{{\sum\limits_{j = 1}^{m} {d_{j} \sigma_{j} } }} $$

where *N*_*j*_ is the number of samples in interval *j*; *N* is the total sample number; *d*_*j*_ is the length of interval *j*; *σ*_*j*_ is the variance of uniformly distributed sampling in interval *j*.

The uncertain flood routing simulation steps for overtopping failure of embankment dam could be described as follows:

1. The random variables affecting the uncertainty of flood evolution and their distribution is determined;
2. Random variables are stratified sampled and corresponding interval probability distributions are calculated;
3. The stochastic process of overtopping failure of embankment dam is numerically simulated adopting stratified sampled variables using HEC-RAS semi-empirical model;
4. The uncertainty transfer model is constructed (flood routing model);
5. The flood risk map of the corresponding sampling variables is obtained by the transfer model, and the normalized weights are assigned according to the distribution probability of the sampled variables;
6. All the sampled risk map calculation results are superimposed to obtain the uncertainty simulation results of the overtopping breach flood of embankment dam.

Under the development environment of Microsoft Visual Studio 2012 and Intel Visual Fortran 2013, FORTRAN90 was used to program and simulate the stochastic process of overtopping dam breach flood routing by invoking the HEC-RAS command flow batch processing program—HEC-RAS Controller. The automatic dam breach simulation and flood routing analysis were realized by repeatedly invoking HEC-RAS Controller. Finally, the calculation results were coded to be output and summarized by QGIS version 3.28^30^.

## Case study

**Figure 2:**
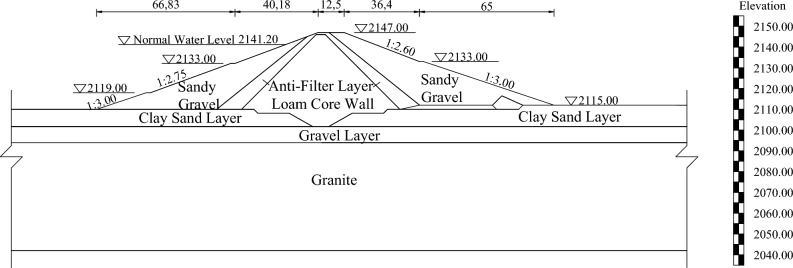
Typical dam section.

A reservoir is located in northwest China with a total storage capacity of 56.44 million square meters. The dam is a loam core dam with a crest length of 150 m, crest width of 12.5 m, maximum dam height of 32.0 m, crest elevation of 2147.00 m. Both upstream and downstream use turf slope protection. The typical dam section is shown in Fig. 2. The dam failed in 1972. According to the follow-up research, the peak discharge of dam breach is approximately 14,800 m^3^/s. The breach formation time is about 1.5 h.

### Calculation model

#### Breach model and related parameters

The BREACH model, MIKE DB model, IWHR DB model and MacDonald equation, Froehlich equation (1995, 2008), Von Thun and Gillete equation, Xu and Zhang equation mentioned above are adopted in overtopping dam failure simulation. Though MIKE DB model and IWHR DB model could not fully simulate dams with core wall, relative studies show that the soil properties are not the very sensitive parameters. The breach process of embankment dam with core wall could be simulated normally as homogeneous dam by using comprehensive parameters representing cohesive soil properties. By regression analysis according to historical dam failure data, the related parameters are shown in Table 1.

**Table 1:** Related dam breach parameters.

Parameters			Value
Dam Shape Parameters	Upstream Slope		2.8
	Downstream Slope		2.7
	Dam Crest Length (m)		150
	Dam Crest Elevation (m)		2147.00
	Dam Bottom Elevation (m)		2115.00
	Reservoir Water Level (m)		2138.57
BREACH Parameters	*D*_50_ (mm)		10
	*D*_90_/*D*_30_		30
	Sandy Gravel	Density (g/cm^3^)	2.20
		Cohesion (kPa)	0
		Internal Friction Angle (°)	34.0
	Loam	Density (g/cm^3^)	2.03
		Cohesion (kPa)	29.4
		Internal Friction Angle (°)	19.0
MIKE DB Parameters	Comprehensive Relative Density		2.65
	Comprehensive Porosity		0.40
	Comprehensive Cohesion (kPa)		22.0
	Comprehensive Internal Friction Angle (°)		20.0
	Critical Shear Stress (MPa)		0.03
	Lateral Erosion Coefficient		0.12
IWHR DB Parameters	Comprehensive Relative Density		2.65
	Comprehensive Porosity		0.40
	Comprehensive Cohesion (kPa)		22.0
	Comprehensive Internal Friction Angle (°)		20.0
	Critical Scouring Velocity (m/s)		2.7
	Weir Flow Coefficient (BCW)		1.44
	Erosion Rate (l/a)		0.91
	Erosion Rate (l/b)		0.0002
Initial and Control Conditions	Final Breach Bottom Elevation (m)		2115.00
	Final Breach Bottom Width (m)		96.0
	Breach Side Slope		1.0
	Initial Breach Elevation (m)		2147.00
	Initial Breach Width (m)		4.0

#### 1D–2D coupled unsteady flow model and related parameters

In this study, HEC-RAS version 6.0 is adopted to simulate 1D–2D coupled unsteady flow.

**Figure 3:**
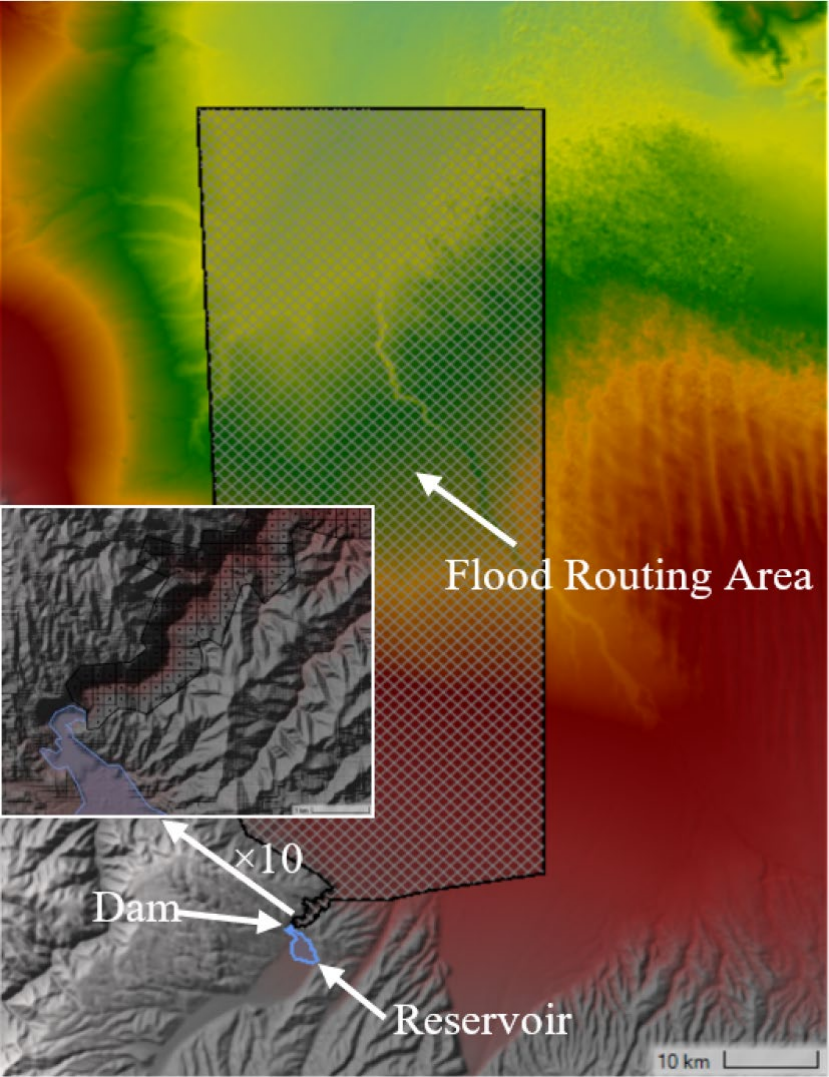
Unsteady flow simulation region.

**Figure 4:**
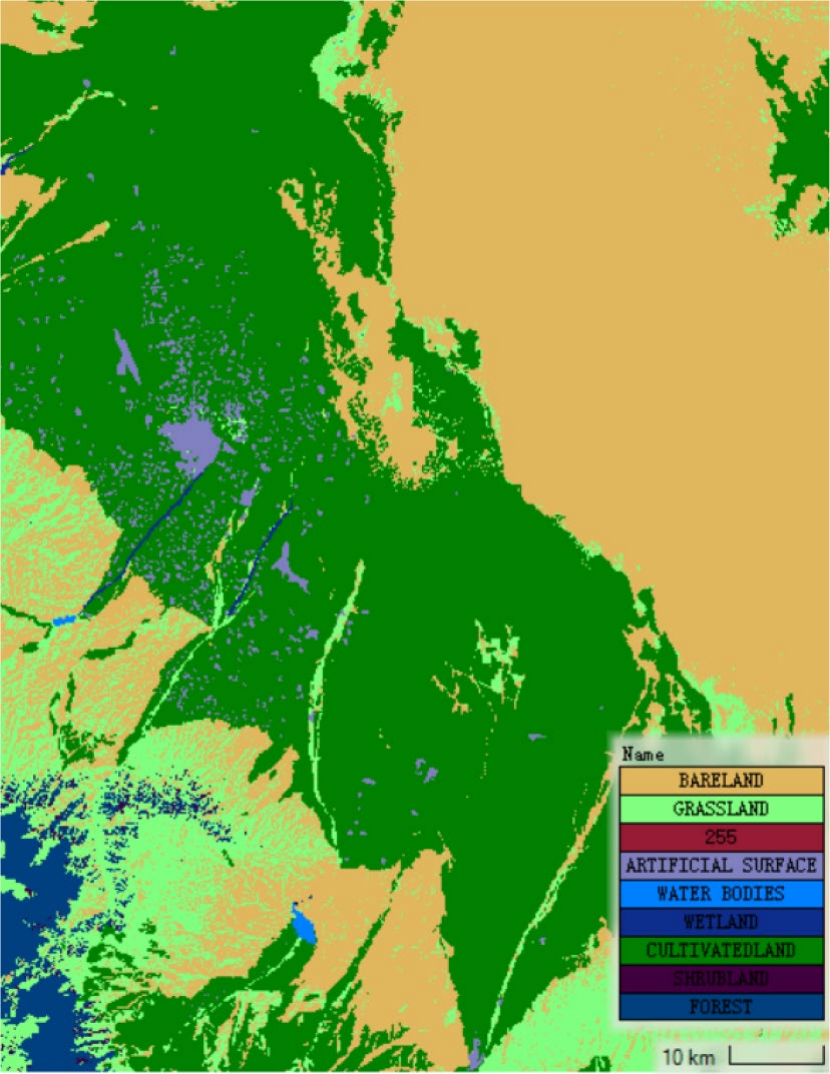
Roughness distribution map^30^.

The reservoir is treated with the linear elevation and storage capacity assumption as a one-dimensional model. The downstream flood routing area is simulated with two-dimensional unsteady flow. The dam is treated as an internal structure boundary between the reservoir and the downstream flood routing area. Topographic elevation data of the flood routing area are obtained from DEM elevation data with 15 m precision. The distribution of Manning roughness coefficient in flood routing area is determined by the land use data provided by GlobeLand. The calculation region is shown in Fig. 3. The roughness distribution map generated by QGIS is shown in Fig. 4. The roughness coefficients are shown in Table 2.

**Table 2:** Topography roughness assignment in flood routing area^11^.

Land type	Roughness (Manning’s *n*)
Bareland	0.020
Grassland	0.035
No Data (255)	0.030
Artificial surface	0.030
Water bodies	0.035
Wetland	0.035
Cultivated land	0.040
Shrubland	0.110
Forest	0.200

#### Boundary conditions

In order to highlight the impact result of uncertainty, the calculation condition is set as follows: when the water level in front of the dam is the flood control level of 2138.57 m and the checked flood occurs, the spillway fails and cannot discharge water normally. The dam break initiates as soon as the reservoir pool elevation reaches dam crest elevation of 2147.00 m. The simulation duration was 24 h including the flood peak.

### Deterministic dam breach simulation results and discussions

**Figure 5:**
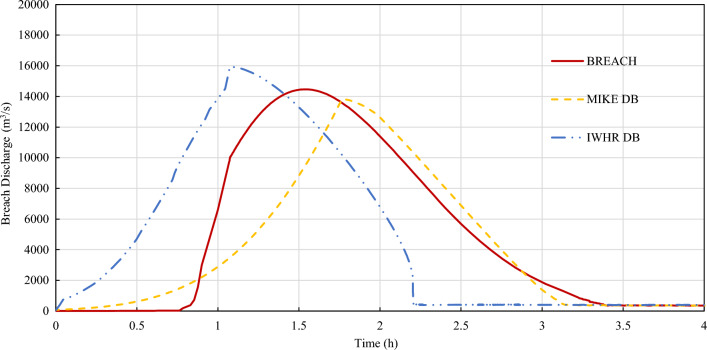
Breach discharge of three commonly used mechanism models.

**Figure 6:**
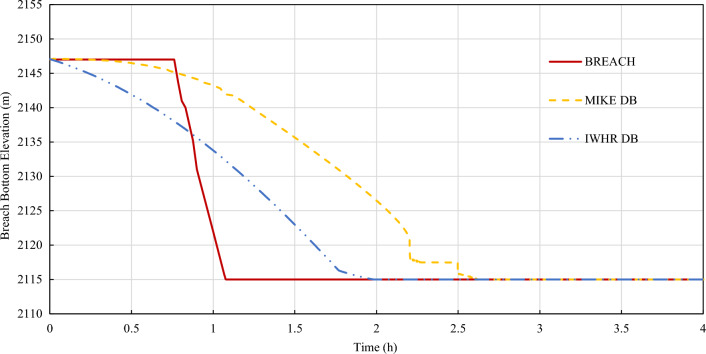
Bottom elevation erosion process of three commonly used mechanism models.

**Figure 7:**
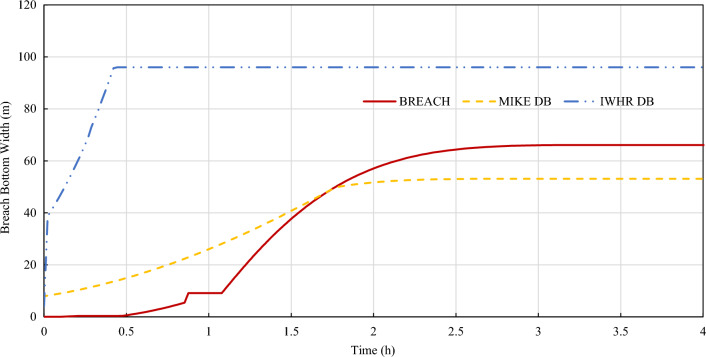
Bottom width erosion process of three commonly used mechanism models.

**Figure 8:**
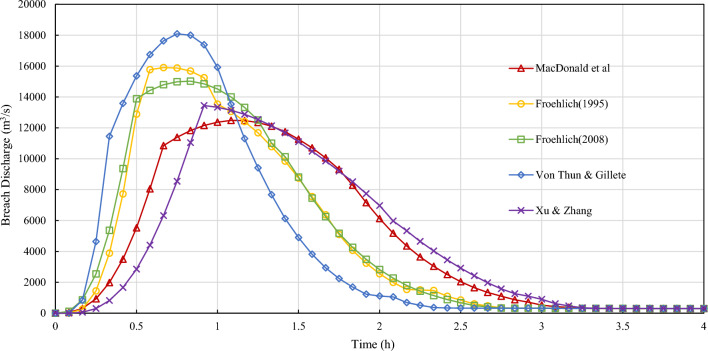
Breach discharge of five commonly used parametric models.

The beginning time of dam breach is defined as time 0. The breach discharge processes of all selected breach models are shown in Figs. 5, 6, 7 and 8. The characteristic values of discharge processes are shown in Table 3.

**Table 3:** Characteristic values of discharge processes.

Breach model		Development time (h)	Peak discharge (m^3^/s)	Peak discharge arrival time (h)	Error of peak discharge relative to mean value (%)
Mechanism model	BREACH	1.08	14,464	0.78	3.50
	MIKE DB	2.19	13,843	1.77	7.64
	IWHR DB	1.79	15,928	1.07	6.27
Parametric model	MacDonald et al.	1.77	12,479	1.08	16.74
	Froehlich (1995)	1.56	15,900	0.67	6.08
	Froehlich (2008)	1.42	15,021	0.83	0.22
	Von Thun and Gillete	0.89	18,086	0.75	20.67
	Xu and Zhang	2.68	13,449	0.92	10.27

According to the calculation results, the erosion processes offered by 3 mechanism models are different. IWHR DB addresses the fastest lateral erosion rate while BREACH has the fastest vertical erosion rate. IWHR DB model tends to first carry out lateral erosion at a fast rate, and then spread vertically. BREACH model occurs sudden acceleration in vertical erosion during slow lateral erosion. The lateral and vertical erosion processes of MIKE DB model are relatively gentle. As a result, MIKE DB model has the smallest peak flow and latest peak flow arrival time.

There are also significant differences between the breach processes offered by 5 parametric models. The results simulated by Xu and Zhang Equations show the longest breach development time and relatively low peak discharge. The results simulated by Von Thun & Gillete Equations show the shortest breach development time and highest peak discharge. The results simulated by Froehlich equations (1995) show the earliest peak discharge arrival time. The results simulated by MacDonald Equations show the latest peak discharge arrival time. The factors of dam breach process in each model are significantly different.

The peak flow and peak arrival time predicted by different models are quite different. The relative mean error of peak flow is up to 7.64% for mechanism models and could reach 20.67% for parametric models. The difference in development time and peak flow arrival time could be more than double. The model uncertainty is significant.

By adjusting the parameter values within a reasonable range and collecting statistics of their impact on the peak breach discharge values, a parameter sensitivity analysis is carried out. The sensitivity percentage and ranking of breach model parameters are shown in Table 5.

The results show that the most sensitive parameters are soil porosity, lateral erosion coefficient and erosion rate (l/b) respectively in BREACH, MIKE DB and IWHR DB models whose sensitivity percentages are all higher than 40%. It could be noted that most sensitive parameters could be controlled in a limited range through site and laboratory test like soil porosity, nonuniform coefficient, internal friction angle, dam slope, et. al. A few parameters have unclear value ranges, like the erosion coefficient in MIKE DB and IWHR DB model. This is a flaw in the model because it is difficult to determine the appropriate value unless there is measured data for calibration. In general, the uncertainty of different models could generally be controlled by the limitation of parameters ranges. The influence of the uncertainty of the breach model is much greater than that of the parameter uncertainty.

### Nondeterministic overtopping dam breach flood routing simulation

#### Stochastic variables and statistics

According to the breach calculation results above, the stochastic variables and their ranges could be generally determined according to Table 4. According to statistics of dam failure events, the final breach bottom elevation usually occurs at 1/3 of the dam height above the dam foundation^22,24,26^. And when the breach bottom elevation is low enough, it is usually not a sensitive parameter as the breach peak flow has occurred before the breach reaches its bottom. Therefore, the final breach bottom elevation is taken as 2125.00–2115.00 m. The broad crested weir formula is used for the open breach. The weir flow coefficient through the breach varies from 1.3 to 1.7 according to relevant studies^13^.

**Table 4:** Breach calculation factors of five commonly used parametric models.

Breach model	Final breach bottom width (m)	Breach slope	Development time (h)	Weir flow coefficient	Final breach bottom elevation (m)
MacDonald et al.	88	0.5	1.77	1.44	2115.00
Froehlich (1995)	110	1.4	1.56		
Froehlich (2008)	96	1	1.42		
Von Thun and Gillete	119	0.5	0.89		
Xu and Zhang	104	1.16	2.68		
Max	119	1.4	2.68		
Min	88	0.5	0.89		

It is assumed that the random variables are independent and normally distributed. According to sensitivity analysis, while considering the adverse impact on security, a large mean square error is adopted for stochastic variables with a large impact on uncertainty and the symmetry axis is biased towards the adverse value. The ranges of parameters that control the breach process are determined according to the parametric model results shown in Table 5. The ranges of roughness coefficients (Manning’s *n*) are determined according to roughness for natural rivers^11^. The random variables used in the nondeterministic simulation and their statistics are shown in Table 6.

**Table 5:** Sensitivity ranking of breach model parameters.

Sensitivity ranking	Breach model					
	BREACH		MIKE DB		IWHR DB	
	Parameter	Sensitivity percentage	Parameter	Sensitivity percentage	Parameter	Sensitivity percentage
1	Soil Porosity	51.80	Lateral Erosion Coefficient	41.28	Erosion Rate (l/b)	40.37
2	*D*_90_/*D*_30_	15.25	Breach Side Slope	14.13	Soil Internal Friction Angle	15.68
3	Soil Internal Friction Angle	7.08	Soil Porosity	11.81	Drop Coefficient of Water Level	10.10
4	Downstream Slope	6.67	Upstream & Downstream Slope	9.38	Soil Cohesion	8.56
5	Core Wall Internal Friction Angle	6.64	Relative Density	5.78	Weir Flow Coefficient	8.05
6	Upstream Slope	5.39	Initial Breach Width	5.61	Critical Shear Stress	7.76

**Table 6:** Stochastic variables and statistics.

Stochastic variable		Mean value	Variable coefficient	Distribution type	Range
Final breach bottom width (m)		103.5	0.2	Normal	88–119
Breach slope		0.95	0.8	Normal	0.67–1.10
Development time (h)		1.13	1.5	Normal	0.89–2.68
Weir flow coefficient		1.44	0.2	Normal	1.3–1.7
Final breach bottom elevation (m)		2118.00	0.01	Normal	2115.00–2125.00
Roughness (Manning’s *n*)	Grassland	0.035	0.8	Normal	0.02–0.05
	Cultivated Land	0.04	0.8	Normal	0.02–0.06
	Bare Land	0.02	1.6	Normal	0.01–0.03

#### Random simulation results of overtopping breach of embankment dam

**Figure 9:**
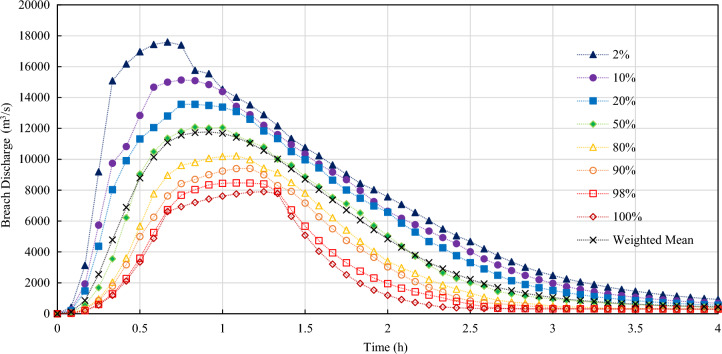
Probability distribution of random discharge process of overtopping breach of embankment dam.

**Figure 10:**
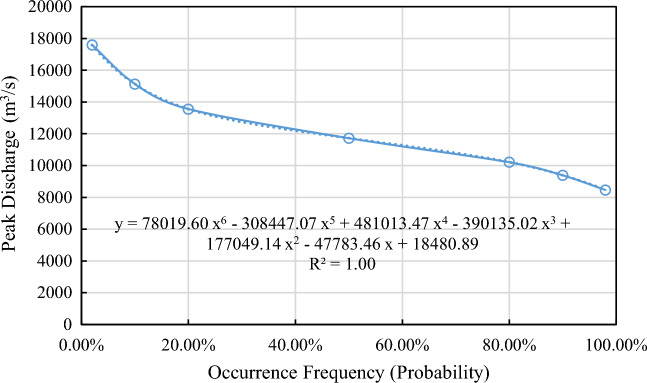
Occurrence frequency (probability) curve of flood peak discharge during overtopping breach of embankment dam.

For the highly sensitive breach bottom elevation and breach development time, five layers of sampling are equally divided to ensure that each interval contains samples. The remaining variables are sampled according to their normal probability distribution with truncation, and a total of 50 groups are sampled. The HEC-RAS semi-empirical overtopping breach model is adopted for stochastic simulation. The results are shown in Figs. 9, 10 and Table 7.

**Table 7:** Results features of random discharge process of overtopping breach of embankment dam.

Probability (%)	2	10	20	50	80	90	98	100
Peak discharge (m^3^/s)	17,593	15,128	13,561	11,728	10,220	9398	8472	7905
Error relative to that of 50% Probability (%)	50.01	28.99	15.63	0.00	− 12.86	− 19.87	− 27.76	− 32.60
Peak discharge arrival time (h)	0.67	0.75	0.83	0.83	1.08	1.08	1.08	1.25

According to the calculation results of random discharge process, the probability of 90% calculated flood peak discharge will not exceed 28.99% of the median peak discharge value. The probability of 80% calculated flood peak discharge will not exceed 15.63% of the median peak discharge value. The lowest peak discharge obtained randomly can also reach about 70% of the median peak discharge value. Therefore, the random discharge process predicted by random overtopping dam breach models is relatively stable. In the extreme case, the peak flow exceeds the median one by 50% (probability: 2%).

#### Random simulation results of flood routing

**Figure 11:**
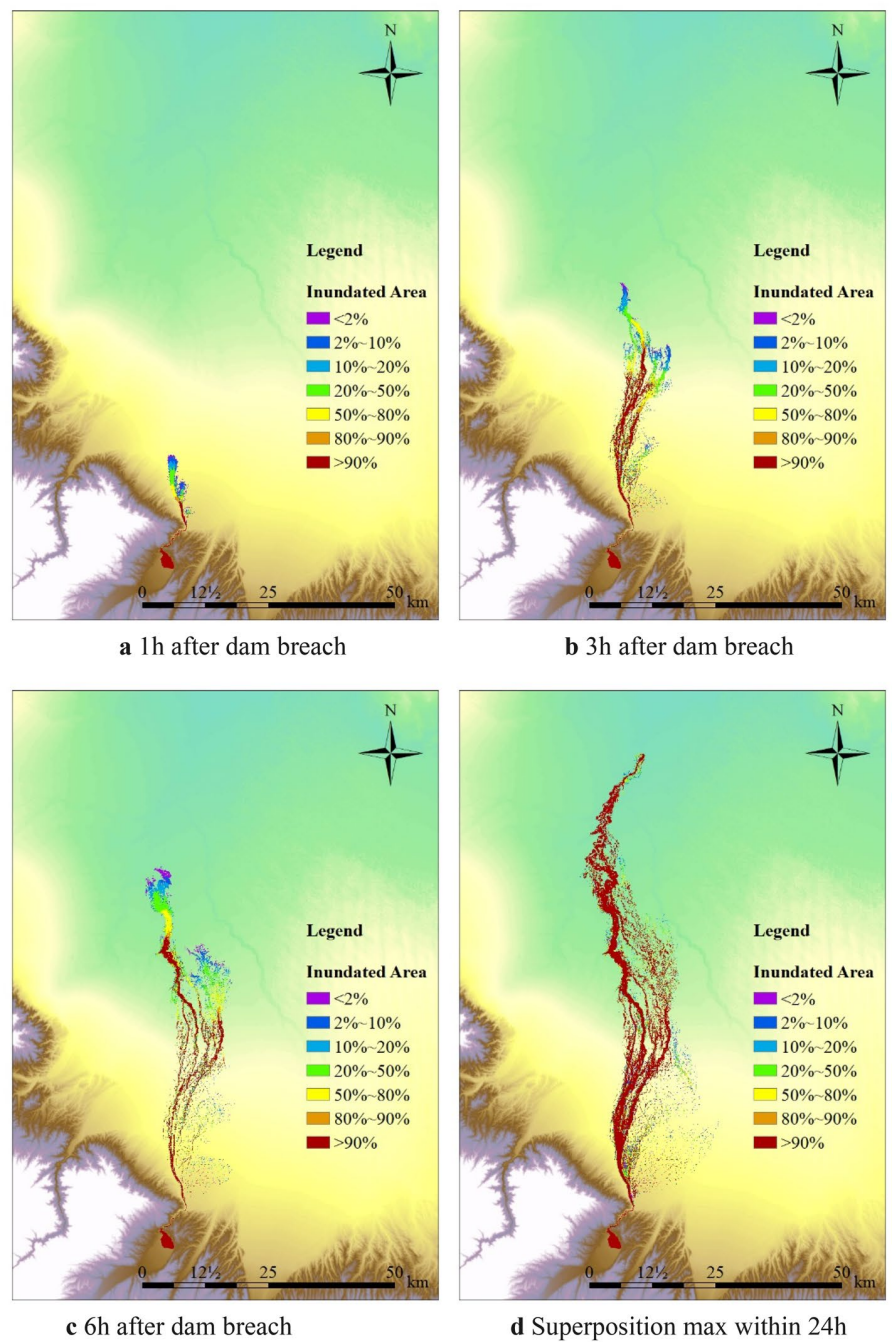
Probability distribution map of inundated area due to dam breach^30^.

The stochastic flood routing is carried out by adopting the random dam breach discharge process as the upper boundary condition. The random simulated flood routing results are weighted and superimposed according to the sampling probability to obtain the uncertain flood routing simulation results. The results maps are generated by QGIS and are shown in Fig. 11. Results features of stochastic overtopping dam breach flood routing are shown in Table 8.

**Table 8:** Results features of stochastic overtopping dam breach flood routing.

Breach time (h)	Inundation statistics	Occurrence frequency (%)							
		2	10	20	50	80	90	98	100
1 h	Main Channel Inundation Length (km)	19.4	19.0	18.4	17.0	14.0	11.4	10.4	10.2
	Length Proportion (%)	100.0	97.9	94.5	87.7	71.9	58.9	53.6	52.7
	Inundated Area (km^2^)	26.7	23.3	20.3	17.3	12.5	10.1	9.4	8.5
	Area Proportion (%)	100.0	87.0	75.9	64.6	46.6	37.9	35.0	31.6
3 h	Main Channel Inundation Length (km)	55.7	54.6	53.7	51.3	46.5	43.2	42.5	42.2
	Length Proportion (%)	100.0	97.9	96.3	92.0	83.5	77.5	76.3	75.7
	Inundated Area (km^2^)	110.0	103.0	98.5	91.4	84.7	82.2	79.8	78.4
	Area Proportion (%)	100.0	93.7	89.6	83.2	77.0	74.8	72.6	71.3
6 h	Main Channel Inundation Length (km)	75.2	72.7	71.9	69.7	65.9	63.4	62.6	62.2
	Length Proportion (%)	100.0	96.6	95.6	92.7	87.7	84.4	83.3	82.7
	Inundated Area (km^2^)	142.1	125.1	114.2	110.9	100.5	97.4	94.4	92.9
	Area Proportion (%)	100.0	88.0	80.4	78.0	70.7	68.6	66.4	65.3
24 h	Main Channel Inundation Length (km)	105.2	105.2	105.2	105.2	105.2	105.2	105.2	105.2
	Length Proportion (%)	100.0	100.0	100.0	100.0	100.0	100.0	100.0	100.0
	Inundated Area (km^2^)	149.7	143.4	139.4	130.6	122.2	119.3	115.3	113.8
	Area Proportion (%)	100.0	95.8	93.1	87.2	81.7	79.7	77.0	76.0
Superposition Max	Main Channel Inundation Length (km)	105.2	105.2	105.2	105.2	105.2	105.2	105.2	105.2
	Length Proportion (%)	100.0	100.0	100.0	100.0	100.0	100.0	100.0	100.0
	Inundated Area (km^2^)	292.1	269.7	259.6	239.6	218.2	209.6	196.3	192.1
	Area Proportion (%)	100.0	92.3	88.9	82.0	74.7	71.8	67.2	65.8

According to the calculation results of stochastic flood routing simulation, during the first hour of flood routing, the flood routing length could vary from 10.2 to 19.4 km. There is only 50% probability allowing the simulated inundated area to reach about 64.6% of the maximum possible inundated area. Only 37.9% of the maximum possible inundated area and 58.9% of the maximum possible inundation length could be reached under the probability of 90%. The minimum inundated area, which is the inevitable inundated range with occurrence frequency of 100%, could only reach 31.6% of the corresponding maximum inundated area and 52.7% of the corresponding maximum inundation length. The results indicate that the uncertainty of flood routing within a few kilometers downstream of the dam is more significant than areas far from the dam.

An hour later, the water moved farther downstream. The minimum inundated area with occurrence frequency of 100% could reach 65% of the corresponding maximum inundated area in each breach period. There is 90% probability allowing the inundated area to reach about 70% of the maximum possible inundated area. At a median frequency of 50%, close to 80% of the maximum possible inundation range could be obtained. Therefore, the simulation results of flood evolution and inundation are relatively controllable within a reasonable range of parameter values. The stability and reliability of the flood routing simulation results could be guaranteed.

In general, though parameters have been controlled in a limited range, the flood routing results in the early stage of dam overtopping failure present greater uncertainty. As the flood progresses further downstream, the uncertainty will gradually decrease. When making flood risk maps and emergency plans, applying conservative value of flood routing simulation parameters will not significantly increase the flood inundation areas far from the dam, and will not cause too much unnecessary waste and loss in emergency scheduling. Therefore, when simulating flood routing for areas far from the dam, it is recommended adopting conservative parameter values. When simulating flood routing for areas near the dam (within about 15 km), adopting appropriate breach models and regression analysis could play an important role in ensuring the accuracy of the results.

## Conclusions

In this paper, an uncertainty analysis on the reliability of flood simulation of overtopping breach of embankment dam is carried out from two aspects: the uncertainty of overtopping dam break and the uncertainty of flood routing. The conclusions could be made as follows:

1. The uncertainty factors in the simulation of overtopping outburst flood of embankment dam mainly come from the uncertain inflow process of reservoir, the uncertain breach process of dam and the uncertain flood routing process. Among them, the uncertain inflow process could be studied and reduced by using conservative frequency analysis value and combined condition strategy. The uncertain breach process of dam is studied by presenting a sensitive study between 3 mechanism breach models and 5 parametric breach models.
2. According to the case study results, IWHR DB addresses the fastest lateral erosion rate while BREACH has the fastest vertical erosion rate. IWHR DB model tends to first carry out lateral erosion at a fast rate which is mainly determined by erosion rate (l/b), and then spread vertically. BREACH model occurs sudden acceleration in vertical erosion when carrying out slow lateral erosion. The lateral and vertical erosion processes of MIKE DB model are relatively gentle. As a result, MIKE DB model has the smallest peak flow and latest peak flow arrival time.

There are also significant differences between the breach processes offered by 5 parametric models. The results simulated by Xu and Zhang Equations show the longest breach development time and relatively low peak discharge. The results simulated by Von Thun and Gillete Equations show the shortest breach development time and highest peak discharge. The results simulated by Froehlich equations (1995) show the earliest peak discharge arrival time. The results simulated by MacDonald Equations show the latest peak discharge arrival time. The factors of dam breach process in each model are significantly different.

The peak flow and peak arrival time predicted by different models are quite different. The relative mean error of peak flow is up to 7.64% for mechanism models and could reach 20.67% for parametric models. The difference in development time and peak flow arrival time could be more than double. The model uncertainty is significant.

The most sensitive parameters are soil porosity, lateral erosion coefficient and erosion rate (l/b) respectively in BREACH, MIKE DB and IWHR DB models. It could be noted that most sensitive parameters could be controlled in a limited range through site and laboratory test like soil porosity, nonuniform coefficient, internal friction angle, dam slope, et. al. A few parameters have unclear value ranges, like the erosion coefficient in MIKE DB and IWHR DB model. This is a flaw in the model because it is difficult to determine the appropriate value unless there is measured data for calibration. In general, the uncertainty of different models could generally be controlled by the limitation of parameters ranges. The influence of the uncertainty of the breach model is much greater than that of the parameter uncertainty.

In general, in the process of nondeterministic dam breach, when the parameters are within a reasonable value range, the influence of model uncertainty on the development and discharge process of dam breach is much higher than that of their respective parameters.

3. By coupling the uncertainty dam breach model with the calculation and analysis method of dam breach flood routing, an uncertainty analysis method for flood routing of overtopping breach of embankment dam is proposed based on stratified sampling Monte Carlo method. The stochastic dam breach process is simulated by HEC-RAS semi-empirical dam breach model. And the flood routing is simulated through HEC-RAS 1D-2D coupled unsteady flow analysis. According to the case study results, though parameters have been controlled in a limited range, the flood routing results in the early stage of dam overtopping failure present greater uncertainty. As the flood progresses further downstream, the uncertainty will gradually decrease. When making flood risk maps and emergency plans, applying conservative value of flood routing simulation parameters will not significantly increase the flood inundation areas far from the dam, and will not cause too much unnecessary waste and loss in emergency scheduling. Therefore, when simulating flood routing for areas far from the dam, it is recommended adopting conservative parameter values. When simulating flood routing for areas near the dam (within about 15 km), adopting appropriate breach models and regression analysis could play an important role in ensuring the accuracy of the results.
4. In this paper, the dam type is embankment dam. Since the breach mechanism and breach model of different dam types are different, corresponding uncertainty analysis and research could be further studied for other dam types on the basis of this paper.

## Data Availability

All associated data have been presented in the manucript which are available from the corresponding author on reasonable request.
